# Dreaming and the neurobiology of self: recent advances and implications for psychiatry

**DOI:** 10.3389/fpsyg.2013.00680

**Published:** 2013-09-26

**Authors:** Armando D'Agostino, Anna Castelnovo, Silvio Scarone

**Affiliations:** ^1^Department of Health Sciences, Università degli Studi di MilanoMilan, Italy; ^2^Department of Mental Health, San Paolo HospitalMilan, Italy

**Keywords:** minimal self, psychosis, schizophrenia, lucidity, dreaming, corollary discharge, sense of agency, self-monitoring

## Introduction: dreams in clinical psychiatry

Throughout most of the 20th century, dreaming was considered at the center of the leading psychotherapeutic approaches to mental disorders. Psychodynamic models of the mind stemmed from Sigmund Freud's Interpretation of Dreams, according to which knowledge of the unconscious foundations of most symptoms could be enhanced by an accurate evaluation of reported dreams. Toward the end of the century, these conceptualizations were challenged by a progressive shift of perspective in the direction of neurobiologically informed, mechanistic models of brain dysfunction. The diffusion of effective psychotropic medications developed to target specific symptoms across different disorders contributed to the requalification of psychiatry itself within the broader domain of medical sciences. A whole generation of psychiatrists turned to biology as a more scientific, rigorous and reliable framework to understand mental disorders. In parallel, new psychological techniques began to emerge that aimed to treat symptoms more directly by addressing dysfunctional cognitive constructs, leading to a relative weakening of the psychodynamic paradigm that is now considered one of several possible models in need of a neuroscientific validation. Neuroscience itself is largely based on cognitive psychology given the major simplicity of its theories in comparison to psychodynamic models, so that neurobiological research paradigms in psychiatry aim to define neural substrates of cognitive mechanisms that differ from the norm (Fusar-Poli and Broome, 2006).

In line with this progression of scientific thought, modern dream research in psychiatry focuses on abnormalities of manifest dream content that can be found within different disorders. Although Post-Traumatic Stress Disorder (PTSD) is the only condition within which specific abnormalities of dream content are considered by clinicians in the diagnostic process—and become a specific target of treatment—several studies have shown that abnormal, disorder-specific dreams are reported by most subjects diagnosed with a mental disorder (Beauchemin and Hays, 1995; Sauteraud and Menny, 1997; Schredl and Engelhardt, 2001; Cartwright et al., 2006; Lusignan et al., 2009). Although several hypotheses on the relationship between dream and waking experiences have been proposed, some of which based on the known abnormalities of sleep rhythms and architecture that occur in psychiatric disorders, no definitive conclusion can be drawn. This difficulty relates to a poor understanding of mechanistic links between psychiatric diagnoses, sleep abnormalities, and chronobiological disruptions that have been recognized and studied for many decades (Wulff et al., 2010).

In this opinion article, we will propose that the progressive refinement of our understanding of dream consciousness could foster significant advances for neuroscience and psychiatry as a whole.

## The dream state: contribution to a general understanding of the brain/mind

Several peculiarities of dreaming make this state a candidate for the experimental study of the brain/mind relationship, a central issue for any possible conceptualization of psychiatric disorders. In recent years, several academics exposed the limits of an excessively operationalized coding system for psychiatric diagnoses (Andreasen, 2007; Craddock and Owen, 2010; Maj, 2011). Structured diagnostic interviews and symptom checklists are known to overestimate comorbidity and often fail to shed light on the core disturbance which causes clinically relevant deterioration of mental health (Maj, 2005). Aside from the obvious behavioral abnormalities that can be observed in some prototypical forms of major disorders, the vast majority of diagnoses depend on clinicians' evaluation of thoughts, percepts, emotions and actions reported by patients. The complexity of these experiences contributes to the construction of a “pathological self” that tends to vary considerably across individuals. In this view, failure to obtain reliable biological markers of any psychiatric disorder could depend on the lack of a clear understanding of the cerebral constituents of the most basic integrative processes that lead to a conscious self in healthy subjects. Although important progress has been made in the fields of neuroimaging and neurophysiology, the question of how any human brain can generate and integrate a self remains elusive and largely open to speculation (Metzinger and Gallese, 2003; Northoff and Bermpohl, 2004; Tononi, 2012).

In this framework, dreaming represents a unique state of consciousness generated when the brain is activated as a closed system, detached from the environment. The dreamer is asleep and unaware of his/her surroundings but develops a full state of consciousness associated with a multimodal reconstruction of dynamic scenes, emotions and interactions with other dreamed people or objects. This is the only physiological, spontaneously recurring state in which complex subjective experiences depend almost exclusively on information stored within the brain. For this reason, dreaming is said to reveal consciousness itself “in a very special, pure, and isolated form” (Revonsuo, 2006).

One relevant limitation to dream research, is the observer's reliance on verbal reports produced by the dreamer upon awakening. As occurs in the context of psychiatric interviews, external observers cannot access the content of subjective experiences directly, but only attempt to reconstruct them on the basis of a narrative. However, the decoding of stimulus- and task-induced brain activity patterns to display visual experiences has already been applied to sleep-onset reports and is likely to be used on complete REM reports in the near future (Horikawa et al., 2013).

## The search for a minimal self

The dream self has been described to largely overlap the core features of the waking self, given its full embodiment and its spatial localization within most of the dream environments (Revonsuo, 2006). Most dream reports involve the presence of a dream self around which the dream itself develops. Indeed, “selfless” dreams are exceedingly rare reports of disembodied centers of awareness that generally occur during Slow Wave Sleep (SWS) (Occhionero et al., 2005). The vast majority of reports suggest that the dreaming brain creates a complex, multimodal hallucinatory reality within which the self is immersed. This hallucinatory immersion has also been recently described as only partially embodied, given the low recurrence of most bodily sensations in dream reports (Windt, 2010). In this state of the brain/mind, self-referential processing is weakened, so that contemporary researchers suggest the search for neurofunctional correlates of what they define as “impoverished selfhood” could by contrast help to clarify the substrate of a minimal self in humans (Blanke and Metzinger, 2009). Such term is used to describe the conscious experience of an embodied subject who is spatially immersed within a first-person perspective. The dream state retains this form of “primary consciousness” associated with an impoverishment of higher order language-related faculties such as self-reflective awareness, abstract thinking, volition, and metacognition termed “secondary consciousness” (Edelman, 1992; Hobson, 2009).

One of the major differences between dreaming and being awake lies in the subject's preserved ability to distinguish real and internally fabricated images only in the latter state of consciousness. Although every human's life is characterized by the brain's continuous distinction between these two modalities, this type of self-reflective ability is only expressed during standard wakefulness. Lucid dreaming and *anoneirognosis*—two rare conditions that have only marginally been characterized in terms of underlying neurobiology—should be considered relevant exceptions with a potential for scientific advancement of knowledge in this field. Table 1 summarizes the major differences between normal dreaming, lucid dreaming and *anoneirognosis*. Whereas lucidity is a physiological condition in which the dreamer acquires secondary consciousness within the dream (Hobson, 2009), *anoneirognosis* is a clinical syndrome of dream-reality confusion associated with defective reality monitoring and executive disorders (Solms, 1997). Both conditions can be interpreted as dissociated REM phenomena, with the latter possibly matching a state of pre-lucidity within which the dream self is doubtful over the origin of the experience. Dissociated phenomena can be understood in terms of intermediate states of consciousness in Allan Hobson's AIM state space model. Advantages of this model for the conceptualization of physiological and pathological transitions of consciousness across the sleep-wake cycle have been discussed elsewhere (D'Agostino and Limosani, 2009).

**Table 1:** **Major differences between normal dreaming, lucid dreaming, and anoneirognosis**.

	**Normal dreams**	**Lucid dreams**	**Anoneirognosis**
Awareness of one's waking self	Absent/Strongly diminished	Preserved	Unknown
		*The dreamer can communicate with external observers through eye-movement protocols memorized during wakefulness.*	
Awareness of one's state (awake/sleep)	Absent	Preserved	Fluctuating
	*The dreamer is fully convinced of being awake. The dreamer can refer to his/her experience as a dream only upon awakening.*	*The dreamer is aware of being asleep. The dreamer can recognize his/her experience as a dream during the dream itself.*	*The subject is often uncertain and fails to distinguish waking experiences from dreams.*
Volitional behavior	Absent/Strongly diminished	Partially retained	Unknown
	*The dreamer mostly acts automatically with no volitional control.*	*The dreamer can at times volitionally control his/her dream.*	

## Dreams and psychosis: from phenomenology to neurophysiology

The similarity between dreams and psychosis has been described by observers belonging to countless philosophical and scientific periods, at least since classical antiquity. Whereas most recent conceptualizations of such analogy attempted to transfer knowledge from the neurobiology of dream sleep to the neuroscience of psychotic disorders (Hobson, 2004; Gottesmann, 2006; D'Agostino et al., 2012), it seems reasonable at the current state of knowledge to consider research in each of the fields could reciprocally contribute to the other (Windt and Noreika, 2011).

The core similarity between these two conditions lies in the subject's inability to discern self-generated from non-self-generated percepts. Indeed, psychotic patients experience hallucinations and construct complex delusional beliefs that grossly impair their social adaptation, without recognizing the internal origin of their experiences. This so-called lack of insight defines the nature of psychosis and persists no matter how bizarre the verbalized experience may appear to an external observer. Likewise, the dreaming subject uncritically accepts the most incongruous and discontinuous twists and turns of the dream plot without ever developing doubts over the nature of his/her experience.

In the prodromal stage of schizophrenia, patients present with a pervasive state of ambivalence and confusion over the meaning of internal and external stimuli which usually leads to the emergence of a unifying delusional explanation. It has been observed that similar to the experience of dreaming, “the subject is confined to his current impressions and perspective at the present moment” (Uhlhaas and Mishara, 2007).

In neurophysiological terms, “corollary discharge” or self-monitoring circuits are known to discriminate sensations dependent on self-generated motor activity from those originating in the environment. According to some authors, such mechanisms are deactivated in the dream state from lower to higher levels of the neuraxis, determining the dream self's loss of a “sense of agency” (Feinberg and March, 1995). Intriguingly, research into loss of the “sense of agency” associated with hallucinations and delusions led to the development of dysconnectivity hypotheses of schizophrenia, according to which disrupted functional connectivity amongst specialized regions is the core neurobiological substrate of the disorder. Indeed, inadequate attribution of the origin of self-produced mental contents could depend on disrupted connectivity within neural networks responsible for sensorimotor integration. Functional neuroimaging techniques confirmed impaired connectivity between prefrontal and temporal cortical structures during language-related tasks and reduced prefrontal activity is commonly associated with failure of self-monitoring in these patients (Stephan et al., 2009). Decreased functional connectivity between brain regions involved in action initiation and perception of its sensory consequences has also been observed in several electroencephalographic (EEG) studies (Ford et al., 2007). Similarly, uncoupled EEG activity between executive and perceptual regions has been experimentally correlated with dreaming during REM sleep (Corsi-Cabrera et al., 2003).

The progression from quiet waking to sleep onset, NREM, and REM sleep, is correlated with an increase in vivid, hallucinatory percepts that are bound together hyperassociatively in bizarre dream experiences. In general, REM dream reports are narrative reconstructions of what the dreamer saw, did, felt emotionally and sometimes thought or said, whereas dreams reported upon awakening from other stages of sleep are thought-like and simple in terms of imagery (Casagrande et al., 1996). In this respect, REM sleep represents a privileged window into a defined neurophysiological state to which a complex pattern of mental activity can be associated. EEG and fMRI studies have shown that the emergence of self-reflection in lucid dreams is associated with a reactivation of frontal cortices that are usually inactive during REM sleep (Voss et al., 2009). The precuneus, a region involved in self-referential processing during wakefulness, was also found to be remarkably more active when REM periods during which lucidity is signaled are contrasted with adjacent REM periods (Dresler et al., 2012).

## Conclusion

Table 2 summarizes some of the most relevant implications of dream research for psychiatry. The growing knowledge in this field will enhance our understanding of how conscious experiences emerge from the activity of the brain. Clearer definitions of the neurobiological bases of a minimal self and of the cerebral underpinnings of self-reflection will shed light on those conditions within which this physiological faculty is lost. Whereas most humans lose this faculty every time they dream and regain it upon awakening, some develop complex mental disorders in which the same loss during wakefulness severely compromises their relationship with the environment.

**Table 2:** **Implications of dream research for psychiatry**.

	**Dream research**	**Psychiatry**
Phenomenology	Definition of dream self in healthy subjects	Epistemological support for a reliable definition of pathological subjective experiences, beyond operationalized diagnoses
Neurobiology	Brain basis of the dream self	Brain basis of altered self-experiences in psychiatric conditions (dissociation, psychosis)
	Brain basis of continuous transitions of subjectivity across the sleep/wake cycle	Brain basis of chronic/acute detachments from reality observed in different psychotic disorders (Schizophrenia, Delusional Disorder, Brief Psychotic Episodes, Mood Disorders with Psychotic features, Substance-related Psychoses)
	Brain basis of lucidity	Brain basis of self-reflective cognition to define the neural underpinnings of psychotic patients' lack of insight
	Brain basis of visual and auditory hallucinatory experiences that occur in dreams	Brain basis of hallucinations that occur in absence of neurological lesions
Pharmacology	Effect of different psychotropic drugs on qualitative and quantitative aspects of dreaming	Neuromodulatory mechanisms underlying specific effects of psychotropic medications

### Conflict of interest statement

The authors declare that the research was conducted in the absence of any commercial or financial relationships that could be construed as a potential conflict of interest.
